# Combination OX40 agonism/CTLA-4 blockade with vaccination reverses anergy and primes tumor-specific CD8 T cells in mice with spontaneous prostate cancer

**DOI:** 10.1186/2051-1426-1-S1-P82

**Published:** 2013-11-07

**Authors:** Stefanie N Linch, Melissa J Kasiewicz, William L Redmond

**Affiliations:** 1 Earle A. Chiles Research Institute, Providence Health Services, Robert W. Franz Cancer Research Center, Portland, OR, USA

## 

Targeted immunotherapy, such as anti-CTLA-4 and anti-PD-1, has proven effective in treating cancer patients. However, despite these advances, cancer remains the second leading cause of death in the US. More effective strategies designed to maximize anti-tumor CD8 T cell responses are necessary to sustain long-term immunity. Agonist anti-OX40 and antagonist anti-CTLA-4 mAb augment the CD8 T cell response through different mechanisms. Therefore, we investigated the additive effects of these modalities on CD8 T cell responses. Combination anti-OX40/anti-CTLA-4 therapy more effectively primed antigen-specific CD8 T cells through enhanced expansion (82% of circulating CD8 T cells) compared to anti-OX40 (61%; P<0.05) or anti-CTLA-4 (41%; P<0.01) alone. Dual therapy also drove expansion of the polyclonal CD8 population. Moreover, combination therapy induced more proliferation (Ki-67) and differentiation (granzyme B, CD127, KLRG-1) following priming, suggesting potent additive effects of these modalities. However, it is known that tumors can induce T cell anergy, thereby limiting an effective anti-tumor response. We tested whether combination therapy could overcome anergy using a mouse model in which mice are tolerant to membrane-bound OVA. Combination anti-OX40/anti-CTLA-4 therapy was uniquely capable of driving robust expansion (12% of total CD8 in the spleen, vs. 2.5% anti-OX40, P<0.05, or 1% anti-CTLA-4, P<0.01) of anergic CD8 T cells, along with enhanced Ki-67 and granzyme B. To further drive expansion of antigen-specific CD8 T cells following therapy, we examined several methods of vaccination using nanoparticles, a Listeria monocytogenes vector, or anti-DEC-205 mAb (all conjugated to OVA) in the presence of anti-CD40. In anergic mice given combination therapy, vaccination with anti-DEC-205-OVA/anti-CD40 produced a 9-fold expansion of OT-I CD8 T cells compared to soluble OVA alone, greater than all other vectors combined. We next tested the efficacy of combination therapy with vaccination on anergy in a spontaneous model of prostate cancer. Combination therapy with vaccination drove expansion (2-fold over monotherapy, P<0.05) and differentiation (granzyme B, KLRG-1, CD25) of both anergic tumor-specific and polyclonal CD8 T cells. Importantly, dual therapy with vaccination enhanced the frequency and number of IFN-γ, TNF-α, and IL-2 producing CD8 T cells. These data indicate that anti-OX40/anti-CTLA-4 combination therapy with vaccination can uniquely augment and drive a robust CD8 T cell response capable of overcoming anergy.

